# A Form of Gonorrhœal Conjunctivitis Not Dependent upon Inoculation

**Published:** 1887-05

**Authors:** 


### A Form of Gonorrhœal Conjunctivitis Not Dependent Upon Inoculation.

The general recognition of the fact that the malignant purulent ophthalmia which occurs with gonorrhœa is due to direct inoculation with the urethral discharge, has often caused it to be forgotten that there is another form of conjunctivitis, which also depends upon gonorrhœa, but is not caused by inoculation. Its existence was recognized by Abernethy, who described cases in which urethritis, articular rheumatism, and an irritative ophthalmia alternated in their occurrence, or in their severity. Most modern writers, however, have looked upon gonorrhœa almost entirely as a local affection, and have ignored the possibility of its affecting the eye, except through direct application of the discharge. In a paper published in Knapp's *Archiv.* (June, 1886), Dr. Haltenhoff, besides quoting cases mentioned by other writers, gives four of his own, in which it seems probable that inoculation played no part. The conjunctival injection is less than in the ordinary cases, and the secretion mucous rather than purulent ; but the most distinctive features are the occurrence of multiple arthritis, the simultaneous affection of both eyes, and the tendency to recur with each attack of gonorrhœa, although, in subsequent attacks, the patient has been careful to shield his eyes from all risk of inoculation. Professor White, of Philadelphia, considers this form of gonorrhœal ophthalmia more common than that induced by inoculation.—*British Medical Journal.*

